# Explainable AI in Digestive Healthcare and Gastrointestinal Endoscopy

**DOI:** 10.3390/jcm14020549

**Published:** 2025-01-16

**Authors:** Miguel Mascarenhas, Francisco Mendes, Miguel Martins, Tiago Ribeiro, João Afonso, Pedro Cardoso, João Ferreira, João Fonseca, Guilherme Macedo

**Affiliations:** 1 Precision Medicine Unit, Department of Gastroenterology, São João University Hospital, 4200-427 Porto, Portugal; francisco.cnm@gmail.com (F.M.); miguel.pedro96@gmail.com (M.M.); tiagofcribeiro@outlook.com (T.R.); joaoafonso28@gmail.com (J.A.); guilhermemacedo59@gmail.com (G.M.); 2 WGO Gastroenterology and Hepatology Training Center, 4200-427 Porto, Portugal; 3 Faculty of Medicine, University of Porto, 4200-427 Porto, Portugal; fonseca.ja@gmail.com; 4 CINTESIS@RISE, Department of Community Medicine, Information and Health Decision Sciences (MEDCIDS), Faculty of Medicine, University of Porto, 4200 427 Porto, Portugal; 5 Department of Mechanical Engineering, Faculty of Engineering, University of Porto, 4099-002 Porto, Portugal; 6 Digestive Artificial Intelligence Development, 4200-135 Porto, Portugal

**Keywords:** artificial intelligence, explainable AI, gastroenterology, gastrointestinal endoscopy

## Abstract

An important impediment to the incorporation of artificial intelligence-based tools into healthcare is their association with so-called black box medicine, a concept arising due to their complexity and the difficulties in understanding how they reach a decision. This situation may compromise the clinician’s trust in these tools, should any errors occur, and the inability to explain how decisions are reached may affect their relationship with patients. Explainable AI (XAI) aims to overcome this limitation by facilitating a better understanding of how AI models reach their conclusions for users, thereby enhancing trust in the decisions reached. This review first defined the concepts underlying XAI, establishing the tools available and how they can benefit digestive healthcare. Examples of the application of XAI in digestive healthcare were provided, and potential future uses were proposed. In addition, aspects of the regulatory frameworks that must be established and the ethical concerns that must be borne in mind during the development of these tools were discussed. Finally, we considered the challenges that this technology faces to ensure that optimal benefits are reaped, highlighting the need for more research into the use of XAI in this field.

## 1. Introduction

As the 21st Century progresses, the revolution associated with the introduction of artificial intelligence (AI) advances into virtually all aspects of our lives. AI applications impact patient care from diagnosis to drug development, allowing for better management and resource allocation, improving healthcare systems’ financial sustainability [1]. Considering digestive health, AI algorithms are offering improvements in endoscopic procedures’ diagnostic accuracies [2]. Moreover, AI models can help predict the risk of disease development, identifying those who benefit from screening or early interventions. Likewise, AI-leveraged data analysis can help tailor treatment plans to each patient. In terms of clinical research, these tools pave the way for identifying novel targets for drug development, potentially accelerating the discovery of new therapies [3]. In addition, AI tools can be used to optimize clinical trial designs, as well as undertaking drug repurposing expeditions [4,5].

One of the main concerns surrounding this AI incorporation is the transparency surrounding its predictions. In healthcare, this is often referred to as “black box” medicine, as opposed to the more readily understood “white box” models, reflecting the difficulties experts may have in understanding and explaining how AI algorithms reach a decision [6]. This commonly results in a loss of trust in decision making, a fundamental aspect of the doctor/patient dynamic [7]. Consequently, the desire for models’ transparency has led to the birth of explainable AI (XAI) and the appearance of “glass box” models that enhance the explainability of AI models without sacrificing their performance [8,9]. XAI is essential to understand how AI tools arrive at their conclusions and the factors that drive their outputs. XAI can help indicate the features that most strongly affect an AI model’s predictions, and it may help explain individual predictions by creating simpler models through a process known as Local Interpretable Model-Agnostic Explanations (LIME) [10]. Conversely, it can evaluate how each feature contributes to a given prediction, providing insights into a model’s reasoning.

In this review, the current state of the art in XAI and its relevance in digestive healthcare were outlined, focusing on its clinical integration, relevant regulatory/ethical considerations, and the technical challenges and associated limitations. Based on this, some speculation will be made as to what awaits this field in the not-too-distant future.

## 2. Foundational Concepts of XAI

The field of XAI focuses on making transparent, interpretable, and accountable models [10]. Transparency implies access to the inner workings of an AI system, including knowledge of the data used in model training, the algorithms employed, and the decision-making process. Interpretability is the ability to understand the reasoning behind AI decisions, with clear and concise explanations of how AI algorithms reach a particular output, which are crucial in identifying any bias or errors in these systems. Accountability refers to the responsibility for the actions and outcomes of an AI system, which are fundamental when deploying AI tools. In addition to these key concepts, model agnosticism must be ensured so that different types of AI models can be employed. AI models must follow a human-centered design, assuring understandable and useful explanations.

Explainability is paramount when AI is employed in sensitive domains like healthcare, where an incorrect diagnosis or inappropriate treatment can have dire consequences [11]. If AI systems make errors, it is essential to understand why this happened to prevent future mistakes. Both healthcare providers and patients need to trust AI systems to gain patient acceptance of AI-driven treatment or diagnoses. Regulatory compliance with the strict regulations regarding data privacy in healthcare is also fundamental. XAI can help demonstrate compliance with these regulations by providing clear documentation of the AI’s decision-making process. Thus, explainability through XAI will be necessary in healthcare to ensure patient safety, build trust, ensure regulatory compliance, and promote ethical AI use.

**Figure 1:**
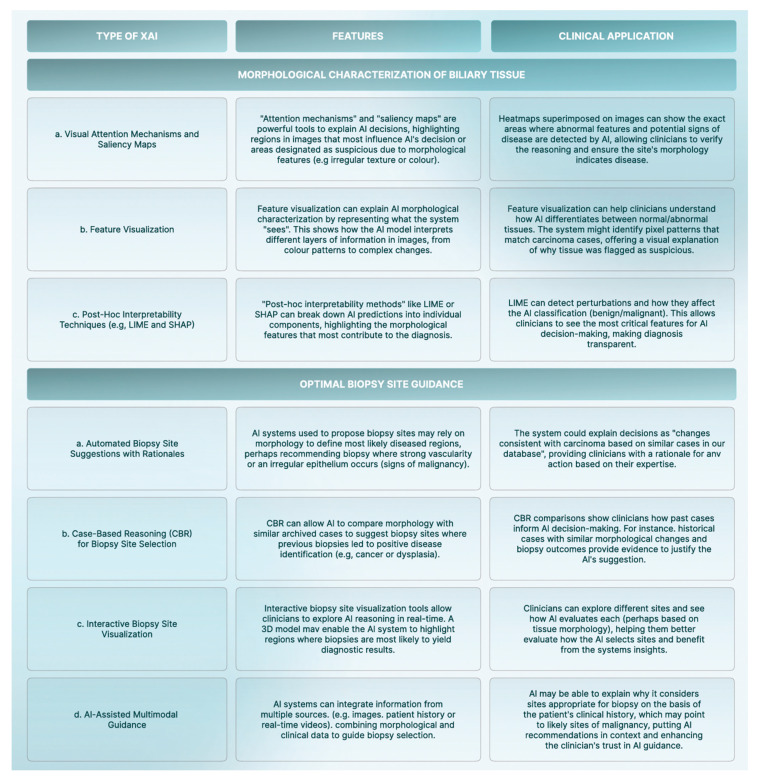
Examples of explainable AI techniques currently available and applicable in digestive medicine to enhance the adoption of AI tools. XAI—explainable AI; AI—artificial intelligence; CBR, case-based reasoning; LIME, Local Interpretable Model-Agnostic Explanations; SHAP, Shapley additive explanations.

The most common XAI techniques used in AI models can be divided into two main categories, those of global or of local explainability, although other approaches also exist (Figure 1). When determining the most appropriate method to adopt, it is important to identify the model type (e.g., linear models, neural networks, and decision trees) as some methods are better suited to different types of models [12]. The degree of explainability required also influences this decision, whether this be global (addressing the overall behavior of the model) or local (focusing on individual predictions). The target audience must be considered to establish the necessary interpretability, while computational demands must also be considered.

## 3. Technological Advances in XAI for Digestive Health

It is clear XAI potentially benefits digestive healthcare, given the potential impact of AI-driven diagnostic and treatment tools [13,14,15]. In terms of explaining individual diagnoses or treatment recommendations, LIME can be used to highlight the most influential factors [16]. By contrast, SHAP (Shapley additive explanations) can provide insights into the contribution of other features to a diagnosis or treatment plan [17,18]. Regarding global XAI techniques, partial dependence plots (PDPs) can be used to visualize how specific features (e.g., age, body mass index, and medication) might influence the probability of a certain disease developing or the effectiveness of a treatment [19]. Accumulated local effects (ALEs) are similar to PDPs but less sensitive to heteroscedasticity, making them more suitable for analyzing complex relationships between different features and outcomes [20].

Firstly, XAI can help clinicians understand how AI-based systems detect abnormalities in endoscopy or radiology exams. XAI can also provide insights into the factors contributing to disease prediction in inflammatory bowel disease or colorectal cancer. Nevertheless, it is fundamental to have effective visualization strategies to understand XAI outputs. Therefore, techniques like heatmaps, feature importance plots, and decision trees can be valuable [21]. Moreover, counterfactual explanations may generate hypothetical scenarios to understand how different patient characteristics affect a diagnosis or treatment recommendation.

AI and ML integration into gastrointestinal (GI) endoscopy is revolutionizing image recognition and analysis. AI algorithms are now available for real-time polyp detection, enhancing exam diagnostic accuracy [22]. AI can also help classify polyps based on size, shape, and color, aiding risk assessment and therapeutic decision making [23]. AI models can offer superior sensitivity in detecting early-stage cancers by analyzing specific image features, prioritizing biopsies and further analysis [24,25]. Indeed, AI can help accurately identify anatomical landmarks in endoscopy images, aiding navigation and procedure planning. The majority of these tools are based on convolutional neural networks (CNNs), which have become the mainstay in endoscopy image analysis due to their performance in terms of feature extraction and classification [26]. Despite these tools’ promise, their clinical validation and integration into routine practice is still challenging.

In this context, LIME XAI can help understand why an AI model differentiates benign and malignant lesions. Moreover, by explaining the features that lead to a cancer diagnosis, SHAP can enhance a clinician’s confidence in the interpretation, potentially leading to earlier detection [27]. Indeed, SHAP improved the performance of an AI model to classify GI tumors, highlighting image features that enhance the interpretability and trustworthiness of the results [28]. An explainable diagnostic approach was also incorporated into an AI model to diagnose gastric cancer, not only enhancing lesion detection but also endoscopist trust and acceptance [29].

**Figure 2:**
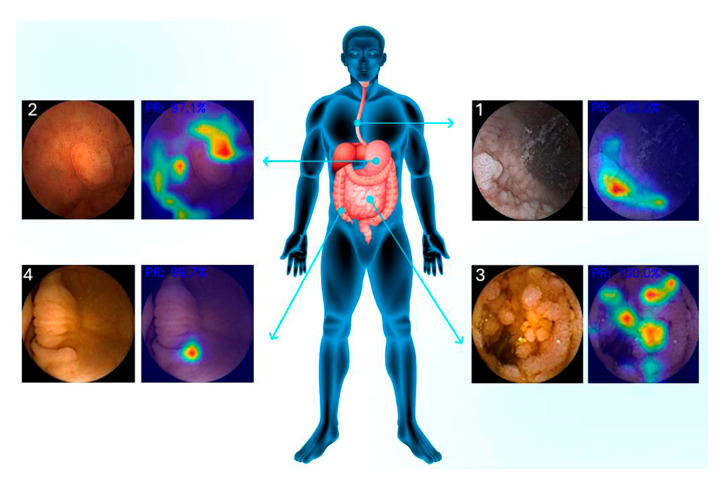
Examples of generated heatmaps for different types of lesions in different locations in Capsule Endoscopy. Each prediction is associated with a degree of certainty expressed with a percentage, while the generated heatmap identified the area responsible for the prediction. The lesions are numbered as follows: 1—P1U—P1 (ulcer lesion by Saurin classification); 2—P1PE (erosion by Saurin classification); 3—PV (vascular lesion). P2V—P2 (vascular lesion); 4—PP/REST (pleomorphic lesion).

Capsule Endoscopy (CE) and Colon Capsule Endoscopy (CCE) are among the leading techniques for which AI tools were generated to analyze large amounts of data [23,30,31]. XAI can provide visual explanations that are crucial for the clinical adoption of these tools, using heatmaps or saliency maps to highlight image regions used to identify a lesion (Figure 2). Explaining why frames are flagged as abnormal helps clinicians trust the decision to focus on relevant images, with XAI providing a brief explanation, resulting in quicker and more accurate diagnoses.

Colonoscopy is widely used to screen and diagnose colorectal diseases, including cancer. AI promises to improve polyp detection rates by providing real-time alerts, where heatmaps or boxes around polyps can highlight features influencing their detection, enabling clinicians to verify findings as true positives [32,33,34]. Surface patterns or vascularity interpretation aid polyp detection and can guide real-time decision making by providing a rationale, such as “the texture and color of this region resemble early-stage adenomas based on training data”.

Upper endoscopy visualizes the esophagus, stomach, and duodenum, helping detect lesions like Barrett’s esophagus, esophageal, or gastric cancers. Saliency maps can highlight suspicious areas and esophageal abnormalities based on abnormal mucosal patterns or color changes [35]. XAI can also provide optimal biopsy site guidance during upper endoscopy, justified through irregular vessel patterns and explaining the features associated with early neoplastic changes [36]. During upper endoscopy, XAI can enhance visualization and provide augmented feedback like overlaid annotations explaining the reason for flagging.

Device-assisted enteroscopy is used to examine the small bowel, where AI can help detect conditions like angiodysplasia, small bowel (SB) tumors, vascular lesions, and inflammatory changes [37]. Heatmaps can highlight regions of interest, such as sites with atypical vascular patterns, villous atrophy, or nodular mucosal changes characteristic of celiac disease or even lymphoid hyperplasia. During therapeutic enteroscopy, XAI can guide optimal interventions and explain the criteria used, helping clinicians make more informed decisions during the procedure.

**Figure 3:**
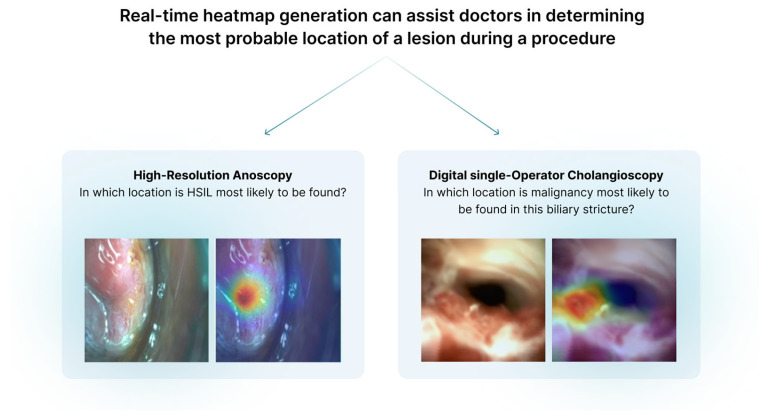
Real-time heatmap generation for lesion location and biopsy guidance in high-resolution anoscopy and digital single-operator cholangioscopy.

Cholangioscopy is the main technique used to diagnose malignant biliary strictures. The difficulty in interpreting subtle morphological changes in cholangioscopy sparked interest in applying XAI, specifically for “morphological characterization” and “optimal biopsy site guidance” (Figure 3) [38,39]. Subtle morphological changes help differentiate benign (inflammation or fibrosis) from malignant (cholangiocarcinoma) biliary strictures, and several XAI approaches can assist in morphological characterization. Malignant biliary strictures are diagnosed through biopsy, with accuracy dependent on selecting the optimal site. XAI can help identify the best biopsy site, enhancing diagnostic accuracy in a transparent and understandable manner. Morphological characterization can be improved through attention mechanisms, feature visualization, or post hoc interpretability techniques while guiding optimal biopsy using case-based reasoning or interactive visualization, improving diagnosis and management [21].

High-resolution anoscopy (HRA) is a diagnostic procedure designed to detect and manage anal intraepithelial neoplasia (AIN) and early anal squamous cancers [40]. The subtle morphological changes associated with dysplasia or early malignancy make HRA challenging, such that AI tools can enhance lesion characterization and biopsy site selection (Figure 2) [41]. To make such tools explainable, visual attention mechanisms and saliency maps can highlight areas considered relevant, overlaying heatmaps of regions with abnormal features (e.g., acetowhite changes, abnormal punctate or mosaic vascular patterns, or irregular lesion borders). Post hoc interpretability methods like LIME or SHAP can help explain the AI assessment, the former perhaps showing texture or color as key factors if AI classifies a region as high-grade squamous intraepithelial lesion (HSIL), the latter providing more detail as to how each feature contributes to the final classification. Optimal biopsy site guidance may be fundamental in HRA, and the techniques available can match features and biopsy outcomes, providing real-time feedback with the identification of areas likely to harbor dysplastic tissue [42]. Through patient stratification based on risk, aligned with lesion severity and characteristics, XAI could recommend monitoring low-grade lesions, treatment of HSIL, or surgical excision of early cancers. With integration of multimodal data (e.g., patient history, human papilloma virus status, and previous treatment responses), a comprehensive analysis can inform treatment decisions [43].

## 4. Integration of XAI in Clinical Settings

XAI’s transparent insights are fundamental to enhance confidence in the diagnoses reached. Clinicians will better understand the rationale behind AI-driven recommendations, identifying potential oversights, with more accurate and comprehensive patient assessments. Similarly, XAI can help identify and mitigate the potential biases in AI models, producing fairer and more equitable patient care.

Additionally, there are significant gains made by accessing patient information through AI-based tools, namely through identifying risk factors that can drive preventive strategies. Coupling these data to diagnostic tests may not only improve diagnostic accuracy but also accelerate decision making to help tailor treatment plans to patients by explaining how their specific characteristics influence treatment outcomes [44].

Moreover, XAI-powered tools have the potential to help automate data entry and analysis, freeing up clinicians’ time for patient care [1]. On the more administrative side, XAI can help identify resource-intensive processes, optimizing the resource allocation of both personnel and equipment.

## 5. Regulatory and Ethical Considerations

Given that the clinical application of XAI-based tools involves accessing sensitive patient data and providing explanations that may reveal patient information, it is important to ensure a well-controlled regulatory framework. Thus, they must comply with the relevant laws and regulations, such as those laid out in the EU General Data Protection Regulation (GDPR 2016/79) or in the USA Health Insurance Portability and Accountability Act (HIPAA). Similarly, it is fundamental to ensure adequate measures to avoid data breaches and unauthorized access. Therefore, most of these regulatory demands must be ensured to avoid conflicts when they are applied in different spheres of influence. Nevertheless, these tools must integrate seamlessly with the technology environments existing in hospitals and clinics, which might pose challenges given the current variation in compatibility and data standards. Indeed, transferability to other settings is an important issue for XAI tools, ensuring they perform equally well in all environments and making it easier to adapt to the rapid changes that are being brought about in their development [45].

Additionally, ensuring that no bias is introduced into the clinical decision-making process is crucial to avoid discriminatory outcomes [46]. If bias is detected, providing understandable explanations is essential to address this issue and avoid introducing further bias. Additionally, it is essential to ensure appropriate human oversight of XAI decisions, despite the difficulty in defining the level of oversight needed. One important ethical issue is identifying responsibility and liability in cases where an XAI system might lead to an incorrect decision being taken, and it remains to be established who is truly responsible, the clinician, the healthcare provider, or the AI developer [47].

## 6. Challenges and Limitations of XAI in Digestive Healthcare

**Figure 4:**
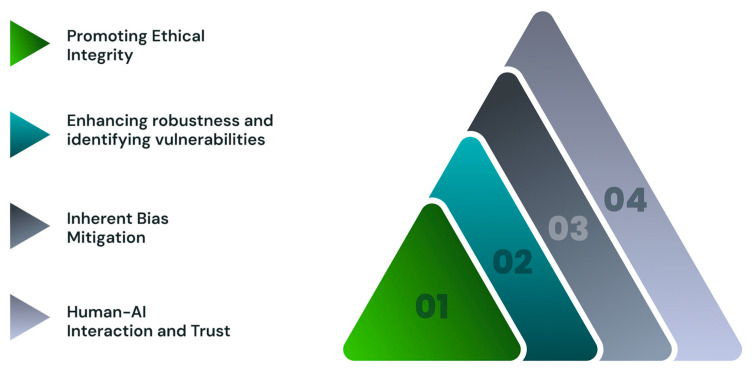
Challenges for implementation of trustworthy explainable artificial intelligence mechanisms in clinical practice.

While there is much promise surrounding the application of XAI in digestive healthcare, a series of challenges must also be met, particularly in terms of data quality and model complexity (Figure 4). The varied data sources in distinct formats and of differing quality make its integration difficult. Moreover, the number of variables may make it difficult to identify the most influential factors, with digestive symptoms being subjective and varying considerably among patients. Balancing interpretability and accuracy are complicated as making XAI models more interpretable may simplify the underlying algorithms and diminish their accuracy. XAI models might also have to provide explanations that can be understood by a variety of people, requiring levels of detail and terminology suited to clinicians, patients, administrators, or other related professionals. Additionally, it will be difficult to evaluate the long-term impact of XAI on patient outcomes and healthcare systems.

Predictive analytics are rapidly transforming healthcare, and gastroenterology is no exception [48]. These models harness data to identify individuals at high risk of disease development, driving earlier diagnosis and treatments [49]. Identifying individuals at risk for conditions like colorectal cancer can support screening programs and lead to recommendations for lifestyle modifications or dietary changes. Similarly, predicting patient response to treatment can improve efficacy and reduce AEs, with better identification of patients that benefit from certain therapies.

While predictive analytics hold immense promise, several challenges must be addressed, such as ensuring that accurate, complete, and standardized data are available for model development, as well as comprehension of factors influencing predictions. Again, patient privacy must be protected and potential biases avoided, and user-friendly tools must be available to seamlessly incorporate predictive analytics into routine clinical practice [2]. Despite these challenges, predictive analytics may provide substantial benefits to gastroenterology, leveraging data-driven insights to drive more proactive and patient-tailored care.

The rapid integration of AI in endoscopy means XAI will be pivotal for their uptake. XAI can provide real-time feedback to endoscopists during procedures, and by explaining the reasons behind AI-generated alerts, it can help prevent mistakes [32,33]. XAI can improve diagnostic tests by identifying sub-optimal images in real time and suggest adjustments to improve visualization. Consequently, patterns in endoscopy data revealed by XAI could offer new insights into disease progression and treatment response, possibly identifying new biomarkers for early disease detection. However, while the rationale behind XAI is to build trust in AI-based tools, perhaps it will first be necessary to build trust in XAI itself. This confidence will only be achieved when the challenges of data privacy, model interpretability, and clinical validation are addressed. However, by overcoming these hurdles, XAI has the potential to revolutionize endoscopy, improving both patient outcomes and procedural efficiency.

XAI has the potential to catalyze cross-disciplinary research and innovation by providing a common language and understanding across different fields [50]. XAI can provide a shared framework for researchers to understand and interpret complex models. XAI can reveal hidden patterns and relationships in data, helping to develop more effective and precise AI models. Nevertheless, it will first be crucial to create a common vocabulary for XAI suited to different fields, as the tools may need to adapt to different specific domains. By addressing these challenges, XAI is likely to become a powerful tool driving innovation and creating new knowledge.

Finally, consideration must be made about the advent of large language models (LLMs) in gastroenterology [51,52]. Initially developed with the purpose of interpreting extensive textual data and answering clinical questions to simplify clinical workflows, recent versions provided the ability to combine textual and image data, ensuring multimodal AI-based analysis [53]. The increased complexity of these LLMs make the comprehension of models’ decisions difficult, reducing both clinician and patient trust. In this context, specific XAI tools must be developed to allow for a better understanding of LLM outputs, with the aim of achieving greater explainability and ensuring clinician trust, a fundamental step for full technology application.

## 7. Conclusions

**Figure 5:**
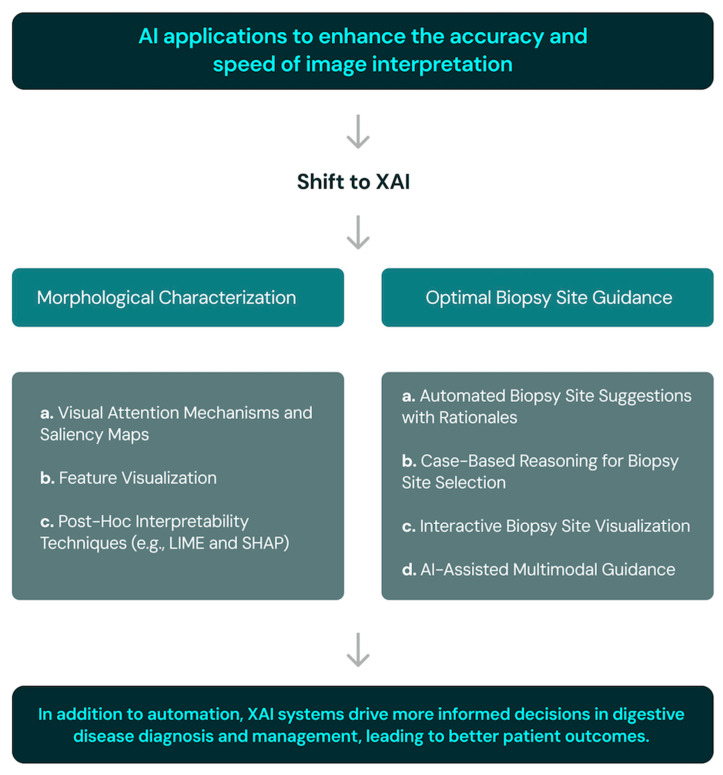
XAI application in digestive medicine. AI—artificial intelligence; LIME—Local Interpretable Model-Agnostic Explanations; SHAP—Shappley additive explanations.

There have been considerable advances in developing AI-based tools for digestive healthcare, but they are often considered black box models, compromising trust in the decisions they reach. XAI was created to address this concern, bridging the gap between complex AI models and human understanding by helping to explain the outcomes of AI models (Figure 5). As these tools aim to provide clear explanations that help clinicians understand AI-generated recommendations, their incorporation is fundamental to build trust in decision making. Indeed, this may be exacerbated by XAI helping to identify potential bias or errors in AI models, understanding the reason behind an incorrect prediction and allowing for ongoing error identification and model improvement, improving the accountability of both the clinician and model developer. Nevertheless, to realize this potential, considerable research is still needed, and efforts to ensure ethical considerations should be adequately considered.

By identifying patient-specific factors influencing treatment outcomes and understanding the factors contributing to disease risk, XAI may be an important motor in personalized medicine. Moreover, with earlier disease detection, more effective interventions and better patient outcomes are expected. Likewise, predicting patient needs through XAI will help optimize resource allocation and reduce waiting times. As XAI offers clinicians the opportunity to avoid black box medicine and preserve their relationship with their patients, patient-friendly XAI tools will better empower them to make informed decisions. While we are still in the early days of XAI implementation in digestive healthcare, there are now examples where these tools can aid in the detection of GI tumors and lesions. Consequently, XAI clearly holds the potential to revolutionize patient care and clinical outcomes.

## Data Availability

No new data were created or analyzed in this study.
